# The Rationale for Angiotensin Receptor Neprilysin Inhibitors in a Multi-Targeted Therapeutic Approach to COVID-19

**DOI:** 10.3390/ijms21228612

**Published:** 2020-11-15

**Authors:** Alessandro Bellis, Ciro Mauro, Emanuele Barbato, Bruno Trimarco, Carmine Morisco

**Affiliations:** 1 Unità Operativa Complessa Cardiologia con UTIC ed Emodinamica-Dipartimento Emergenza Accettazione, Azienda Ospedaliera “Antonio Cardarelli”, 80131 Napoli, Italy; abellis82@vodafone.it (A.B.); ciro.mauro1957@gmail.com (C.M.); 2 Dipartimento di Scienze Biomediche Avanzate, Università FEDERICO II, 80131 Napoli, Italy; emanuele.barbato@unina.it (E.B.); trimarco@unina.it (B.T.)

**Keywords:** COVID-19, neprilysin, natriuretic peptide, angiotensin II, bradykinin, apelin, substance P, adrenomedullin, sacubitril/valsartan

## Abstract

The severe acute respiratory syndrome coronavirus 2 (SARS-CoV-2) disease (COVID-19) determines the angiotensin converting enzyme 2 (ACE2) down-regulation and related decrease in angiotensin II degradation. Both these events trigger “cytokine storm” leading to acute lung and cardiovascular injury. A selective therapy for COVID-19 has not yet been identified. Clinical trials with remdesivir gave discordant results. Thus, healthcare systems have focused on “multi-targeted” therapeutic strategies aiming at relieving systemic inflammation and thrombotic complications. No randomized clinical trial has demonstrated the efficacy of renin angiotensin system antagonists in reducing inflammation related to COVID-19. Dexamethasone and tocilizumab showed encouraging data, but their use needs to be further validated. The still-controversial efficacy of these treatments highlighted the importance of organ injury prevention in COVID-19. Neprilysin (NEP) might be an interesting target for this purpose. NEP expression is increased by cytokines on lung fibroblasts surface. NEP activity is elevated in acute respiratory distress syndrome and it is conceivable that it is also high in COVID-19. NEP is implicated in the degradation of natriuretic peptides, bradykinin, substance P, adrenomedullin, and apelin that account for prevention of organ injury. Thus, NEP/angiotensin receptor type 1 (AT1R) inhibitor sacubitril/valsartan (SAC/VAL) may increase levels of these molecules and block AT1Rs required for ACE2 endocytosis in SARS-CoV-2 infection. Moreover, SAC/VAL has a positive impact on acute heart failure that is very frequently observed in deceased COVID-19 patients. The current review aims to summarize actual therapeutic strategies for COVID-19 and to examine the data supporting the potential benefits of SAC/VAL in COVID-19 treatment.

## 1. Introduction

The outbreak of severe acute respiratory syndrome coronavirus 2 (SARS-CoV-2) has become a major concern all over the world. The disease induced by SARS-CoV-2 is named COVID-19. It refers to an interstitial pneumonia with distinctive vascular features, consisting of severe endothelialitis associated with the presence of a wide cellular injury [1]. The central role of endothelial damage in the pathogenesis of COVID-19 is confirmed by the frequent involvement of the cardiovascular system in an early stage of the disease, as reflected by the release of highly sensitive troponin and natriuretic peptides (NPs) [2].

It is always more recognized that the pathogenicity for COVID-19 is enhanced by an inflammatory overreaction leading to abnormal production of cytokines to fight the viral infection [3]. This phenomenon is called cytokine release syndrome (CRS). Hence, many studies targeted the utilization of some immune-modulatory agents as COVID-19 therapies to minimize the disease severity [4]. Simultaneously, identifying angiotensin-converting enzyme 2 (ACE2) as a viral entry receptor emphasized the important role of the classical renin–angiotensin–aldosterone system (RAAS) in COVID-19 pathophysiology. Some researchers suggested that the use of ACE inhibitors and/or angiotensin receptor blockers (ARBs), may blunt the severe inflammatory reactions and improve endothelial dysfunction caused by stimulating angiotensin II type 1 receptors (AT1Rs) [5]. Interestingly, one RAAS component, namely neprilysin (NEP), is implicated in the degradation of molecules exerting a protective effect on lung and cardiovascular system. Moreover, NEP has emerged as an interesting pharmaceutical target for treatment of cardiovascular disease, in particular of heart failure (HF) [6,7], that is a frequent lethal consequence of SARS-CoV-2 infection [2].

The current review aims to summarize actual therapeutic strategies for COVID-19 and to examine the data supporting the potential benefits of NEP inhibition in COVID-19 treatment.

## 2. COVID-19 Pathophysiology

SARS-CoV-2 is closely related to SARS-CoV. In fact, they both use ACE2 as the receptor-binding domain for their spike (S) protein, which is formed by two subunits (S1 and S2) [8]. The S1 subunit features the receptor binding domain that interacts with ACE2. Host cell infection can be blocked by a clinically proven inhibitor of the cellular transmembrane protease serine 2 (TMPRSS2), which is required for S protein priming of both coronaviruses [8]. Virus binding induces ACE2 endocytosis and AT1R plays an important role in this phenomenon for SARS-CoV infection [9]. Probably, this also works for SARS-CoV-2 infection. Furthermore, antibody responses raised against SARS-CoV S protein could at least partially protect against SARS-CoV-2 infection [8]. Thus, it is conceivable that SARS-CoV and SARS-CoV-2 share the same pathogenetic mechanism through affecting ACE2 activity.

Notably, ACE and its close homologue ACE2, exert two opposite physiological functions. ACE cleaves angiotensin I (Ang I) to generate angiotensin II (Ang II), the peptide which binds to and activates AT1Rs to constrict blood vessels, thereby elevating blood pressure. Conversely, ACE2 inactivates Ang II while generating angiotensin 1–7, an heptapeptide with a potent vasodilator function, thus serving as a negative regulator of the RAAS. The binding of the SARS-CoV S protein to ACE2 leads to ACE2 down-regulation and to a lower production of angiotensin 1–7 [10]. The latter results into higher Ang II concentration that contributes to increased pulmonary vascular permeability mediated by AT1R in animal models [10].

It has been postulated that unabated Ang II activity may be also in part responsible for organ injury in COVID-19 [5]. Such hypothesis is consistent with the very recent finding that ACE2 gene disruption in a murine model determines a spontaneous corneal inflammation through a cytokine storm-like mechanism [11]. Interestingly, this phenotype could be partially rescued by treatment with the ARB losartan, thereby suggesting that the observed effect was mediated by Ang II acting on its main receptor. Moreover, as a consequence of higher Ang II levels, an increase in systemic cytokines, especially interleukin-6 (IL-6), has been observed in subjects with COVID-19 [12]. This corresponds to the characteristics of a CRS [13] and CRS development in COVID-19 is associated with COVID-19 severity.

## 3. Actual Therapeutic Strategies for COVID-19

### 3.1. Remdesivir

A wide variety of antivirals are currently being evaluated in clinical trials. Among these, remdesivir gained priority for inclusion in COVID-19 clinical studies because of its broad-spectrum activity against human and zoonotic coronaviruses in pre-clinical models. Unfortunately, the data that emerged from all randomized clinical trials (RCTs) investigating the efficacy of remdesivir against placebo among patients with COVID-19 are discordant. In the first RCT, there was no significant difference in time to clinical improvement between the treatment (200 mg of remdesivir on day 1 followed by 100 mg on days 2–10 in single daily infusions) and placebo groups (the same volume of placebo infusions for 10 days) [14]. Conversely, it has been successively reported that remdesivir (200 mg loading dose on day 1, followed by 100 mg daily for up to 9 additional days) was superior to placebo in shortening the time to recovery in hospitalized COVID-19 patients with evidence of lower respiratory tract infection [15]. More recently, it has been demonstrated that only patients randomized to a 5-day versus 10-day course of remdesivir (200 mg loading dose on day 1, followed by 100 mg daily for up to 4 or 9 additional days in two treatment arms, respectively) significantly clinically recovered compared to those treated with standard care, but this difference was of uncertain clinical importance [16].

The discordance in results from RCTs was confirmed by two published metanalyses. In the first one, it was observed that both the 5-day course and the 10-day course of remdesivir were associated with significantly better clinical improvement than placebo, while the 5-day course of remdesivir was associated with significantly better clinical improvement than the 10-day course of remdesivir [17]. Nevertheless, in this analysis, the clinical improvement within 10–15 days after randomization was measured with an ordinal scale, whereas clinical criteria and information about supportive treatments (high-flow or low-flow supplemental oxygen therapy, invasive or non-invasive mechanical ventilation, extracorporeal membrane oxygenation) were not considered. Conversely, the second metanalysis indicated the presence of mortality benefits within 14 days of administration of remdesivir, but an absence of mortality benefits within 28 days of the administration, with no difference in 14-day mortality between 5-day and a 10-day course of remdesivir [18]. This apparent discrepancy between outcomes was explained by the authors by the fact that COVID-19 patients surviving past 14 days without remdesivir treatment are probably able to recover to a similar extent to those receiving treatment with remdesivir.

Thus, in the waiting for more conclusive results about the efficacy of selective anti-SARS-CoV-2 agents, healthcare systems have focused on “multi-targeted” therapeutic strategies aiming at relieving systemic inflammation and preventing thrombotic complications.

### 3.2. Drugs Fighting Systemic Inflammation

Some authors suggested that withdrawal of RAAS inhibitors may be harmful in high cardiovascular risk patients with known or suspected COVID-19, because these drugs are able to counteract the inflammatory state induced by unabated Ang II stimulation in SARS-CoV-2 infection [5]. Consistently, based on a large-scale retrospective study, in-hospital use of ACE inhibitors/ARBs was associated with a lower risk of 28-day death among hospitalized patients with COVID-19 and coexisting hypertension, coronary artery disease and hypertension combined with coronary artery disease [19]. Nevertheless, to date, no clinical randomized trial has validated the efficacy of these drugs in preventing or even reducing COVID-19’s severity.

Plasma from patients healed from SARS-CoV-2 was used to block Fc Fragment Receptor activation for the urgent treatment of pulmonary inflammation [20]. Such a treatment may be combined with systemic anti-inflammatory drugs or corticosteroids. In particular, the use of selective interleukin-6 receptor (IL-6R) antagonist tocilizumab has been demonstrated to reduce the risk of invasive mechanical ventilation or death in patients with severe COVID-19 pneumonia [21]. The administration of dexamethasone resulted in lower 28-day mortality among patients receiving either invasive mechanical ventilation or oxygen alone at randomization [22]. However, the proper timing of administration and the true dose of tocilizumab are still unknown and need to be addressed by studies currently underway. Consistently, due to their lack of benefit and potential harm among patients who did not require oxygen, indications for dexamethasone have to be better defined.

The pharmacological modulation of molecular pathways accounting for cytokine release may also have relevance in COVID-19 patients. In particular, chloroquine (CQ) and its less toxic metabolite hydroxychloroquine (HCQ), affect the release of tumor necrosis factor-alpha (TNF-α), IL-1 and IL-6 in viral infections by flaviviruses, retroviruses, and coronaviruses [23]. However, although a pre-clinical evidence considering CQ effectiveness for treatment of COVID-19 showed promising results in a small Chinese cohort, data from clinical studies with CQ/HCQ have been not satisfactory [24].

Because of their immune-modulatory effect, chronic statin use was associated with a lower risk of developing severe COVID-19 and a faster time to recovery among patients without severe disease [25]. Nevertheless, this is an observational and relatively small sample size study which cannot prove causality.

### 3.3. Antithrombotic Therapy

The application of heparin in COVID-19 has been recommended because of the risk of disseminated intravascular coagulation and venous thromboembolism leading to pulmonary embolism. It is also known that heparins have several non-anticoagulant properties, and can exert anti-inflammatory effects. In fact, heparins block P-selectin, the cross-talk of platelets and neutrophils [26], inhibit neutrophil response and the formation of neutrophil extracellular traps (a phenomenon called NETosis) [27], and reduce the release of IL-1β, IL-6, and of adhesion molecules [28,29]. Furthermore, heparins determine the release of extra-cellular superoxide dismutase (EC-SOD) from endothelial cells’ surface, thereby increasing plasmatic concentrations of this enzyme [30]. EC-SOD is principally implicated in counteracting the deleterious effects of reactive oxygen species (ROS) on the functions of both pulmonary cells and red blood cells observed in the most severe cases of COVID-19 [31]. Interestingly, there is evidence of a link between decreased expression of the antioxidant enzyme SOD3 in the lungs of elderly patients with COVID-19 and disease severity [32]. Thus, increased EC-SOD plasmatic concentrations by heparin treatment could balance the excess of ROS and prevent sudden deterioration of clinical conditions in more frail COVID-19 patients.

In addition to their anti-coagulant and anti-inflammatory properties, heparins are under investigation for potential use as direct antiviral agents due to their inhibitory effects on pathogen adhesion to cell surfaces. The direct antiviral effect of heparins involves the heparan sulfate, a family of polysaccharides, ubiquitous components of the cell surface and extra-cellular matrix of all animals [33]. Heparan sulfate has been known to work as the initial point of contact between target cells and several human viruses, including SARS-CoV-2 [34]. Heparins have been shown to efficiently compete with heparan sulfate and, by that, attenuate viral attachment and cell infection. Moreover, it has been recently reported that the SARS-CoV-2 S1 receptor binding domain is bound by heparin and that, upon binding, a significant structural change is induced, providing evidence for a direct antiviral effect [34]. Finally, several TMPRSSs, such as cathepsins, factor Xa, furin and trypsin, have been shown to proteolytically process the S protein of SARS-CoV-2. In particular, the factor Xa has been shown to facilitate the activation of SARS-CoV entry into the cells [35]. Because all heparins are inhibitors of several proteases like factor Xa, thrombin, furin and cathepsin-L [36], it may be hypothesized that this could be another direct mechanism to avoid cellular entrance of SARS-CoV-2.

Unfortunately, data for the potential of heparin to reduce mortality in COVID-19 are limited. In particular, anticoagulant therapy, mainly with low-molecular-weight heparin, appears to be associated with better prognosis in COVID-19 patients with a high risk of sepsis-induced coagulopathy or with a markedly elevated D-dimer [37]. A second observational study showed that therapeutic anticoagulation was associated with longer survival among critically ill patients with COVID-19 [38]. However, both these observational studies had important limitations and highlighted the need for randomized trials. To this purpose, the design for an international, open-label, adaptive randomized controlled trial has been recently proposed [39].

## 4. Controversial Role of NEP in COVID-19

The still discordant results about efficacy of actual therapeutic strategies renders the prevention of lung and heart injury as a topical issue in the conceptualization of a “multi-targeted” approach to COVID-19 treatment. In fact, acute respiratory distress syndrome (ARDS) and cardiovascular injury are both significantly and independently associated with COVID-19 mortality [40]. In this context, the zinc metallopeptidase NEP, widely expressed in endothelial cells, smooth muscle cells, fibroblasts, and cardiomyocytes, seems to play a controversial role. 

On one side, it has been recently postulated that increasing NEP activity may mitigate COVID-19 pathogenesis [41]. In fact, this enzyme has been reported to have more catalytic activity than ACE2 in cleaving the vasoconstrictor peptides Ang I and II to vasodilator peptide Ang 1–7 [42]. Furthermore, NEP can play a key role during lung inflammation through its catabolic effect on gastrin-releasing peptide (GRP) [43]. GRP is one of the bombesin-like peptides that can be expressed and released by the pulmonary neuroendocrine cells into the surrounding airway parenchyma in response to hypoxia or irritation in order to induce the neutrophil chemotaxis and enhance macrophage infiltration within the lung tissue [44]. Thus, breaking GRP by NEP will prevent recruitment of more neutrophils into the site of injury. NEP can additionally decrease the pro-inflammatory, oxidative and pro-fibrotic effects of Ang II by minimizing the release of cathepsin G and its catalytic action on Ang I [45].

**Figure 1:**
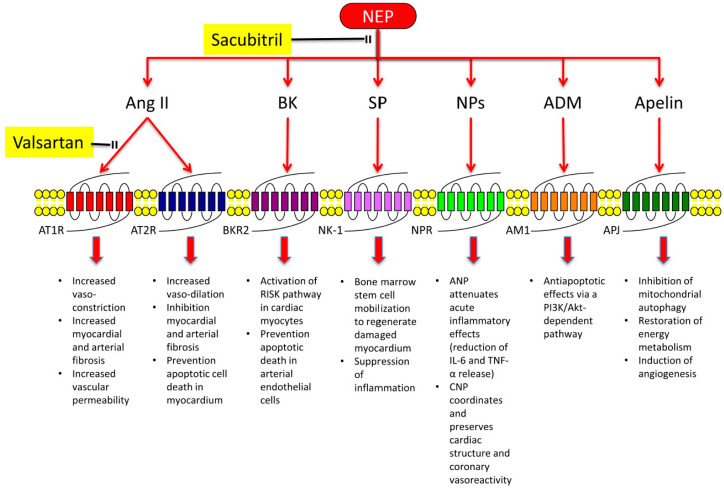
Effects of angiotensin receptor and neprilysin (NEP) inhibition. Angiotensin receptors type 1 (AT1Rs) inhibition by valsartan reduces vasoconstriction, myocardial fibrosis, and vascular permeability induced by angiotensin II (Ang II). Consistently, it favours vasodilation and cell protection against apoptotic death through angiotensin receptors type 2 (AT2Rs). NEP inhibition by sacubitril increases levels of natriuretic peptides (NPs), bradykinin (BK), substance P (SP), adrenomedullin (ADM), and apelin, thereby amplifying protective pathways mediated by these molecules. BKR2: bradykinin receptor 2; NK-1: neurokinin-1 receptor; NPR: natriuretic peptide receptor; AM-1; adrenomedullin receptor-1; APJ: apelin receptor; RISK: reperfusion injury salvage kinase; IL-6: interleukin 6; TNF-α: tumor necrosis factor-α.

By the other side, NEP catalyses the degradation of vasodilator peptides, including NPs, bradykinin (BK), substance P (SP), and adrenomedullin (ADM), as well as of apelin isoforms (Figure 1) [46,47]. Interestingly, NPs have been found to reduce the release of inflammatory mediators, such as IL-6 and TNF-α, in experimental model of acute lung injury [48]. Furthermore, most of NEP substrates account for cardiovascular protection. In fact, atrial NP (ANP) attenuates acute inflammatory effects of mast cells or histamine [49] and C-type NP (CNP) regulates and preserves cardiac structure, function, and coronary reactivity via activation of NP receptor-C (NPR-C) [50]. BK activates reperfusion injury salvage kinase (RISK) pathways in cardiomyocytes [51] and significantly reduces apoptotic death induced by prolonged hypoxia in arterial endothelial cells [52]. SP induces bone marrow stem cell mobilization that is able to suppress inflammation in ischemic heart [53]. ADM cardioprotective power is mainly attributed to antiapoptotic effects via a phospatidyl-inositol-3 kinase (PI3K)/Akt-dependent pathway [54]. Finally, the apelin/APJ system decreases cardiovascular injury through the inhibition of mitochondrial autophagy [55], restoration of energy metabolism [56], and induction of angiogenesis [57].

**Figure 2:**
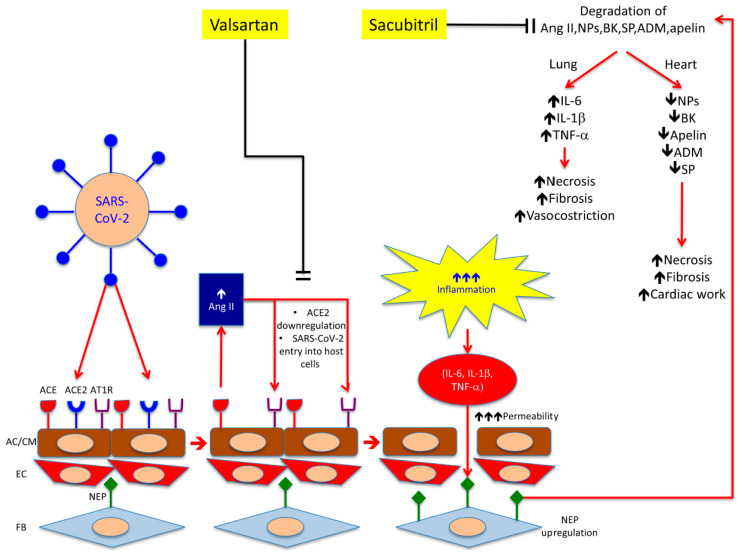
Working hypothesis for sacubitril/valsartan therapy in COVID-19. In the lung, severe acute respiratory syndrome coronavirus 2 (SARS-CoV-2) binds and down-regulates ACE2 on alveolar cell surface. AT1R antagonism (valsartan) induces a compensatory Ang II synthesis by ACE, but also reduces ACE2 and SARS-CoV-2 endocytosis. Consistently, inhibition of up-regulated NEP (sacubitril) on fibroblasts surface affects NPs degradation that have been found to reduce the release of inflammatory mediators (IL-6 and -1β, TNF-α). Lowering cytokine levels might decrease alveolar permeability and risk of ARDS onset. In the heart, sacubitril accounts for a prolonged NPs activity and results in reduction of cardiac work through increased diuresis. Sacubitril might also play a direct protective effect against apoptotic cardiomyocyte death through inhibition of BK, SP, ADM, and apelin degradation. AC: alveolar cells; CM: cardiomyocytes; EC: endothelial cells; FB: fibroblasts; ACE: angiotensin converting enzyme; ACE2: angiotensin converting enzyme 2; Ang II: angiotensin II; AT1R: angiotensin 1 receptor; NEP: neprilysin; NPs: natriuretic peptides; BK: bradykinin; SP: substance P; ADM: adrenomedullin; IL-6 and -1: interleukin 6 and -1. Red arrows indicate activation pathways; black lines indicate inhibition pathways; ↑ indicate a marked increase ↑↑↑ indicate a marked increase; ↓ indicates a decrease.

On the basis of these considerations, we may hypothesize that, during SARS-CoV-2 infection, the clinical picture of ARDS is worsened by an abnormally increased cytokine production at pulmonary level that is less antagonized because NP degradation is mediated by NEP up-regulation in lung fibroblasts. The following “cytokine storm” might induce NEP up-regulation in other districts, especially the heart and endothelium, thereby increasing the degradation of protective compounds (NPs, BK, SP, ADM, and apelin) that, in turn, could account for the systemic severe effects of COVID-19 (Figure 2).

**Figure 3:**
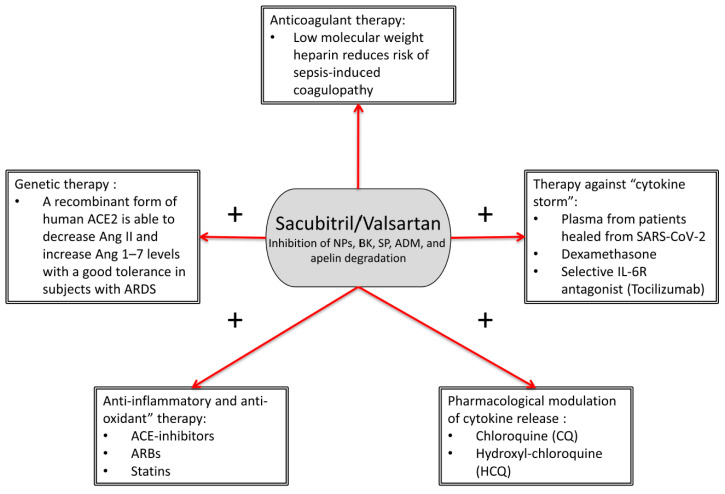
Sacubitril/valsartan (SAC/VAL) in a “multi-targeted” therapeutic strategy for COVID-19. SAC/VAL could have potential relevant synergistic effects with most of drugs commonly used in COVID-19 patients, thereby more efficiently fighting the organ injury induced by SARS-CoV-2 infection. Sign “+” indicates pharmacological associations with potential synergistic effects. NPs: natriuretic peptides; BK: bradykinin; SP: substance P; ADM: adrenomedullin; ARBs: angiotensin receptor blockers; ARDS: acute respiratory distress syndrome.

Therefore, we think that in a “multi-targeted” therapeutic strategy for COVID-19, where the inflammatory burden is antagonized by specific anti-inflammatory drugs such as corticosteroids (dexamethasone) and tocilizumab, NEP antagonism becomes relevant for prevention of organ injury (Figure 3).

## 5. Rationale for Angiotensin Receptor Neprilysin Inhibition in COVID-19

Sacubitril/valsartan (SAC/VAL) is a well-tolerated NEP/AT1R inhibitor (ARNI) providing concomitant antagonism of NEP (via LBQ657, the active metabolite of the prodrug sacubitril) and blockade of AT1Rs (via valsartan). It has been successfully used in the treatment of chronic [6] and acute decompensated HF [7], and it is under investigation in the setting of acute myocardial infarction as anti-remodelling agent (PARADISE MI study; NCT02924727). The adoption of SAC/VAL has been recently proposed in COVID-19 patients [58]. However, the rationale of this proposal is restricted to the anti-inflammatory effects of SAC/VAL. Conversely, we think that there are further reasons for supporting SAC/VAL as a viable approach to prevent adverse effects of SARS-CoV-2 infection.

### 5.1. Pathobiology of Comorbidities Associated with COVID-19

A wide variety of important histological lesions have been observed during autopsy of patients who died from SARS-CoV-2 infection (Table 1) [59]. Moreover, several studies have reported a higher prevalence of cardiovascular disease including hypertension, obesity, and diabetes in hospitalized patients with COVID-19, and there is evidence that these comorbidities are associated with disease progression to hypoxemia, ARDS, and death [60].

In the United States, there has been a disproportionately high rate of COVID-19-related hospitalizations and death among older individuals and African Americans [60]. Both populations have a high degree of salt sensitivity and salt-sensitive hypertension [61]. The pathobiology underlying hypertension and salt sensitivity in these populations may be implicated in their vulnerability to COVID-19-induced ARDS. One key factor that associated with salt sensitivity is impaired secretion of ANP. Plasma ANP levels, which increase in normotensive subjects fed a high-salt diet, paradoxically decrease in black hypertensive subjects in response to a high-salt diet [62]. In the Dallas Heart Study, hypertension was more prevalent, whereas unadjusted N-terminal pro-brain natriuretic peptide (NTproBNP, a precursor of BNP) levels were lower, in black than in white and Hispanic individuals [63]. This striking relationship between COVID-19 disease severity and NPs levels can also be found in obese and diabetic individuals. It is well established from epidemiological studies that circulating NPs levels are lower in obese individuals [64]. Similarly, NT-proBNP is inversely associated with risk of incident diabetes [65].

Thus, dysregulation of NPs pathway seems to be associated with more severe COVID-19 and it is conceivable that increasing NPs levels by SAC/VAL administration could be beneficial to counteracting the adverse effects of SARS-CoV-2 infection.

### 5.2. Reduction of Lung Injury

In an experimental and clinical study of ARDS, the activity of NEP was significantly increased in the alveolar air space, thereby suggesting a pivotal role of this enzyme in acute lung injury [66]. In fact, it has been demonstrated that several cytokines, including IL-1α, TNF-α, transforming growth factor, IL-6, and granulocyte macrophage colony-stimulating factor, enhance activity of NEP on the surface of intact lung fibroblasts [67]. Increased NEP activity may reduce levels of NPs, that play an important protective role in the lungs. In fact, ANP was discovered to block thrombin-induced increases in endothelial permeability [68] and to attenuate lung endothelial permeability caused by various insults, including oxidant stress, lipopolysaccharide (LPS), and TNF-α [69]. Furthermore, ANP reduced the secretion of inflammatory mediators in response to LPS in macrophages [70]. Finally, ANP infusion improved arterial oxygenation and lung injury score in patients with ARDS during mechanical ventilation [71].

Therefore, inhibition of NPs degradation by SAC/VAL might play a protective role in the lung during SARS-CoV-2 infection. 

### 5.3. Reduction of Heart Injury

The mechanisms underlying myocardial injury during COVID-19 remain unknown. In particular, it is still unclear whether acute injury is a primary infective phenomenon or secondary to lung disease. Two principal hypotheses have been formulated [72]. The first one accounts for a direct injury of myocardium (myocarditis), and the second one for a precipitation of a pre-existing atherosclerotic disease. In the last case, several pathways associated with viral diseases may contribute to forming and destabilizing atherosclerotic plaques (type 1 myocardial infarction) in COVID-19 patients [73]. Nevertheless, it is important to consider that type 2 myocardial infarction is the most common subtype in viral conditions, probably determined by the infection of pericytes leading to severe microvascular dysfunction in non-obstructive coronary arteries. Both myocarditis and precipitation of atherosclerosis are mediated by CRS leading to HF, cardiac arrhythmias, rapid deterioration, and sudden death [74].

SAC/VAL might account for cardiovascular protection through the reduction in endothelial cells and cardiomyocyte apoptosis by affecting NPs, BK and apelin isoforms’ degradation in SARS-CoV-2-infected patients (Figure 2) [51,52]. Interestingly, recent studies suggest that intracellular Ang II may also exert protective effects in cardiomyocytes and endothelium, during high extracellular levels of this hormone following NEP inhibition. Ang II, through nuclear AT1Rs, promotes protective mechanisms by stimulation of the AT2R signalling cascade [75]. In particular, the stimulation of nuclear Ang II receptors enhances mitochondrial biogenesis, thereby protecting the heart against oxidative stress [75]. Thus, despite abundant data about the deleterious effects of Ang II on the heart, a growing body of evidence suggests a protective role for this molecule that could be of relevance in supporting the hypothesis of SAC/VAL use in prevention of cardiovascular injury in COVID-19.

### 5.4. HF Therapy

It is remarkable that acute HF is the consequence of a wide myocardial injury and one of the most frequently observed complications (49% of cases) in deceased COVID-19 patients [2]. Given the beneficial effects on the prognosis of acute HF patients, it is conceivable that SAC/VAL might have a positive impact [7]. Unfortunately, an association between SAC/VAL assumption and prognosis improvement in HF patients affected by COVID-19 has not been yet investigated.

### 5.5. Antagonism of SARS-CoV-2 Endocytosis

SARS-CoV-2 is closely related to SARS-CoV. In fact, they both use ACE2 as the receptor-binding domain for their S protein [8]. Virus binding induces ACE2 endocytosis and AT1R plays an important role in this phenomenon for SARS-CoV infection [9]. This likely also works for SARS-CoV-2 infection.

SAC/VAL blocks AT1R, which is required for ACE2 endocytosis (Figure 2). This effect might antagonize SARS-CoV-2 entry into the host cells, thereby reducing the extension of infection.

### 5.6. Pharmacodynamics, Safety and Limitations

SAC/VAL is not in contrast with other drugs commonly used in COVID-19 therapy. Because of its inhibitory effect on degradation of atrial NPs leading to a reduction in IL-6 release, SAC/VAL might have a synergistic effect with IL-6R antagonist tocilizumab (Figure 3). Consistently, dedicated drug interaction studies demonstrated that the co-administration of therapy for HF (such as furosemide, carvedilol, amlodipine, omeprazole, hydrochlorothiazide, and atorvastatin) did not affect systemic exposure to SAC/VAL [76].

Nevertheless, we have to keep in mind that there are also some limitations to SAC/VAL use. As an example, hypotension is a very frequent potential collateral effect of this therapy and SAC/VAL is not to be used if blood systolic pressure is below 100 mmHg [76]. However, the right SAC/VAL dosage for each patient may be found through the progressive titration to the highest tolerated quantity of the drug, according to blood pressure values, in order to reduce the risk of hypotension [77]. Furthermore, concomitant administration of potassium-sparing diuretics (e.g., the mineralocorticoid receptor antagonist spironolactone, also used as an anti-ventricular remodelling agent) [78], potassium supplements, or salt substitutes containing potassium may lead to an increase in serum potassium concentrations. In patients who are elderly, volume-depleted, or with compromised renal function, SAC/VAL may result in possible acute renal failure [76].

## 6. Clinical Perspectives and Conclusions

The observed benefit of in-hospital ACE inhibitors/ARBs’ use compared to non-ACE inhibitors/ARBs drugs in COVID-19 patients is consistent with the anti-inflammatory effects of both these pharmacological classes [19]. SAC/VAL can exert an additive action, because it may increase levels of protective compounds (NPs, BK, SP, ADM, and apelin) against lung and heart injury.

Firstly, we propose to analyse the impact of a previous SAC/VAL therapy on COVID-19 severity. We expect a reduced severity of COVID-19 in this cluster of patients. If this is the case, the following step could be a randomized-controlled study aimed to test the effect of SAC/VAL addition to standard therapy on mortality and on other secondary endpoints (hospitalization, duration of in-hospital stay, worsening of symptoms, evidence of multi-organ failure, etc.) in moderate COVID-19. The causative relation between SAC/VAL administration and prognosis improvement could be validated by relief of increased urinary cGMP, which is a good detector of the biological effect of SAC/VAL on NPs-mediated activation of NPs receptors [79], in COVID-19 patients with a better outcome.

In the absence of selective antiviral therapies, healthcare systems have focused on “multi-targeted” therapeutic strategies aiming at relieving systemic inflammation and preventing thrombotic complications in COVID-19 patients. The still-controversial efficacy of these treatments highlighted the importance of organ injury prevention. In this context, the ARNI SAC/VAL could play a relevant role. Future randomized trials will be required to support this hypothesis.

## Figures and Tables

**Table 1:** Most important histological lesions observed during autopsy of patients who died from SARS-CoV-2 infection and related hypothesized pathophysiological mechanisms. * indicates the histological lesions recognizing the direct viral infection as pathophysiological mechanism; ^§^ indicates the histological lesions recognizing a pathophysiological mechanism other than the direct viral infection.

Organ	Histological Lesions	Pathophysiological Mechanisms
**Lung**	Diffuse alveolar damage * Focal vasculitis and capillaritis associated to microthrombosis * Thrombosis of large and medium size pulmonary arteries ^§^	* Direct viral effect ^§^ SARS-CoV-2-associated coagulopathy or deriving from the deep veins of the lower estremities
**Heart**	Myocarditis * Ischemic myocardial injury (atherosclerotic plaque activation or increased coronary reactivity) ^§^	* Direct viral effect ^§^ SARS-CoV-2-associated cytokine storm and coagulopathy or pericytes infection by SARS-CoV-2 (MINOCA)
**Kidney**	Acute tubular injury mainly involving the proximal tubules	Probably related to direct infection of kidney by SARS-CoV-2
**Skin**	Urticarial rashes and papulovesicular exanthems Livedoid purple lesions and acrocyanosis Kawasaki disease	Cause not yet known
**Central Nervous System**	Aspecific acute hypoxic damage in the brain and cerebellum	Molecular positive test for the virus, but negative immunohistochemistry (also consider SARS-CoV-2-associated coagulopathy)
**Liver**	Sinusoidal dilatation with lymphocytic infiltration and steatosis	Cause not yet known
**Adrenal**	Acute fibrinoid necrosis of arterioles	Cause not yet known
**Testis**	Seminiferous tubular injury, mild lymphocytic inflammation	Cause not yet known
